# Italian Candidates for the XEN Implant: An Overview from the Glaucoma Treatment Registry (XEN-GTR)

**DOI:** 10.3390/jcm11185320

**Published:** 2022-09-09

**Authors:** Chiara Posarelli, Michele Figus, Gloria Roberti, Sara Giammaria, Giorgio Ghirelli, Pierpaolo Quercioli, Tommaso Micelli Ferrari, Vincenzo Pace, Leonardo Mastropasqua, Luca Agnifili, Matteo Sacchi, Gianluca Scuderi, Andrea Perdicchi, Romeo Altafini, Maurizio Uva, Dino D’Andrea, Giuseppe Covello, Maria Novella Maglionico, Antonio Maria Fea, Carmela Carnevale, Francesco Oddone

**Affiliations:** 1 Department of Surgical, Medical, Molecular Pathology and of Critical Area, University of Pisa, 56124 Pisa, Italy; 2 IRCCS Fondazione Bietti, 00198 Rome, Italy; 3 Ospedale San Pietro, Fatebenefratelli, 00186 Rome, Italy; 4 Ospedale Generale Regionale F. Miulli di Acquaviva delle Fonti, 70021 Bari, Italy; 5 Ophthalmology Clinic, Department of Medicine and Aging Science, University G. D’Annunzio of Chieti-Pescara, 66100 Chieti, Italy; 6 University Eye Clinic, San Giuseppe Hospital, University of Milan, 20162 Milan, Italy; 7 Ophthalmology Unit, NESMOS Department, S. Andrea Hospital, Faculty of Medicine and Psychology, University of Rome La Sapienza, 00189 Rome, Italy; 8 Ospedale di Dolo, 30031 Venice, Italy; 9 Azienda Ospedaliera Universitaria, “Policlinico Vittorio Emanuele”, P.O. Gaspare Rodolico, 95123 Catania, Italy; 10 Ospedale Dell’Aquila, 67100 L’Aquila, Italy; 11 Ophthalmic Eye Hospital, Department of Surgical Sciences, University of Turin, 10122 Turin, Italy

**Keywords:** glaucoma, surgical treatment, XEN gel, outcomes, real-life, registry

## Abstract

**Background** The Italian XEN Glaucoma Treatment Registry (XEN-GTR) was created to acquire a comprehensive prospective dataset that includes the patient characteristics, intraoperative variables, and postoperative management of glaucoma patients undergoing the XEN gel stent implantation. **Methods** This was a prospective observational, longitudinal clinical study involving 10 centres throughout Italy. The baseline examination included a comprehensive evaluation of demographic parameters (age, sex, ethnicity, and systemic condition), specific ophthalmological parameters, and quality of life questionnaire score collection. **Results** The baseline data of 273 patients were analysed. The median (IQR) age was 72 (65.0 to 78.0) years. Of the 273 patients, 123 (45%) were female and 150 (55%) were male. A total of 86% of the patients had open-angle glaucoma with a mean intraocular pressure of 24 ± 6 (range 12.0–60.0) mmHg. The mean number of medications was 2.7 ± 0.9 at baseline for the patients with a prevalence of prostaglandin analogues combined with a beta-blocker and anhydrase carbonic inhibitor (31.8%). The mean scores of the NEI-VFQ 25 and GSS questionnaires were 78 ± 18 (range 26.5–100) and 85 ± 14 (range 79–93), respectively. Combined XEN/cataract surgeries were scheduled in 73.7% of the patients. The preferred place for the XEN implant was the supero-nasal quadrant (91.6%). **Conclusions** Observing the baseline characteristics of the typical Italian candidates for the XEN gel implant shows that they are patients affected by POAG and cataracts, with moderate to severe glaucoma damage, all of which has an impact on their quality of life.

## 1. Introduction

Real-world observational studies have become increasingly important in providing evidence of treatment effectiveness in clinical practice. While randomized clinical trials are the gold standard for evaluating the safety and efficacy of new therapeutic strategies, necessarily strict inclusion and exclusion criteria mean that trial populations might limit the generalizability of their results and are often not representative of the target patient population encountered in clinical practice.

Moreover, real-world studies can provide information on the long-term safety and effectiveness of treatments in heterogeneous populations [1], as well as information on utilisation patterns and health and economic outcomes. 

It has been well established that glaucoma is associated with a reduction of quality of life (QoL), and that QoL decreases with advancing disease severity [2]. Increased attention to QoL has emerged in recent years and new surgical techniques have been designed to reduce the possible morbidity connected with traditional glaucoma surgery [3,4]. One promising technique involves the use of the XEN gel implant (XEN, Allergan, Dublin, Ireland), which is a biocompatible 6 mm gelatine microtube designed to create a channel from the anterior chamber to the subconjunctival space and to allow aqueous humour outflow, and it has a minimally invasive procedure when inserted through a small corneal incision [5,6,7,8,9].

The purpose of the Italian XEN Glaucoma Treatment Registry (XEN-GTR), an observational, prospective, longitudinal clinical study, is to obtain a comprehensive prospective dataset that includes patients’ characteristics, intraoperative variables, and postoperative management patterns for analysis of the effectiveness, safety, quality of life, and cost-effectiveness outcomes of the treatment. 

The baseline characteristics of the first 273 Italian patients enrolled are described herein and could represent an overview of the typical Italian candidates for the XEN implant.

## 2. Materials and Methods

### 2.1. Study Design and Patient Population

This is a prospective, observational study involving 10 centres throughout Italy. Consecutive patients diagnosed with glaucoma and deemed to be suitable for the XEN implant, according to the normal indications and procedures of the attending ophthalmologist, were considered for inclusion if the following criteria were met: age > 18 years, ability to understand and sign written informed consent, diagnosis of open-angle glaucoma according to the diagnostic criteria of the 5th edition of the Guidelines of the European Glaucoma Society [10], and an indication for treatment with the XEN implant, in the opinion of the ophthalmologist treating the patient, such as glaucoma progression or uncontrolled IOP despite medical treatment. Patients who needed cataract surgery in combination with glaucoma surgery were also included as patients with primary angle closure glaucoma, with indications for the XEN45 implant, only if it was combined with phacoemulsification.

The exclusion criteria included the following: secondary glaucoma (different from pseudoexfoliation and pigmentary glaucoma); the presence of conjunctival scarring or conjunctival pathologies (e.g., pterygium) in the target quadrant; signs of active inflammation (e.g., blepharitis, conjunctivitis, keratitis, or uveitis); active iris neovascularization or the presence of iris neo-vessels within 6 months of the date of surgery; intraocular lenses in the anterior chamber; the presence of intraocular silicone oil; vitreous humor in the anterior chamber; altered episcleral venous drainage (e.g., Sturge–Weber syndrome or nanophthalmos); known or suspected allergy or sensitization to drugs required for the surgical procedure or for some component of the device (e.g., glutaraldehyde or porcine derivatives); and a history of keloid scars. If a patient needed surgery in both eyes, only the data of the first eye were included in the register.

The baseline clinical data were collected before surgery (1 to 4 weeks before surgery, at visit 0) and included the demographic and ophthalmologic variables listed in Table 1. The data collected on the day of surgery (visit 1) were strictly related to the procedure and to the surgeon’s habits (Table 1).

Eligible patients, after signing a written informed consent form, underwent a comprehensive eye examination and medical and ocular history evaluation. The eye examination included a best-corrected visual acuity (BCVA) assessment, a slit lamp evaluation, a gonioscopy, an IOP measurement using Goldmann applanation tonometry (the average of 3 measurements was considered for the analysis), and an indirect dilated ophthalmoscopy with a 90 dioptres lens. 

A visual field test (standard white-on-white perimetry), retinal nerve fibre layer thickness optical coherence tomography (OCT), and an endothelial cell count were also performed. 

The endothelial cell count was performed by means of non-contact specular microscopy, and the average of 5 measurements was considered for the study purposes.

During the visit, patients were also asked to complete the Italian version of two questionnaires: the NEI-VFQ-25 and the GSS [11,12].

### 2.2. Statistical Analysis 

Continuous data are described as means and standard deviations if normally distributed or as medians and interquartiles if not normally distributed. Categorical and nominal data are described as frequencies.

## 3. Results

Between January 2018 and October 2020, 273 patients were enrolled.

One hundred and fifty patients were male (55%), while one hundred and twenty-three patients were female (45%). The median (IQR) age was 72.0 (65.0 to 78.0) years, with a frequency of comorbidities such as hypertension (47.6%, 130/273), diabetes (13.6%, 37/273), and autoimmune diseases (5.9%, 16/273). Anticoagulant therapy was being undertaken by 16.5% (45/273) of the patients. 

Baseline ophthalmological data are reported in Table 2 and Table 3. According to perimetry classification [13], 105/273 (42.7%) patients were classified as having severe glaucoma (mean defect of worse than −12 dB), 66/273 (26.8%) as having moderate glaucoma (mean defect of between -6 to −12 dB), and 58/273 (23.6%) as having early glaucoma (mean defect of better than −6 dB), respectively. The data of 44 patients (16.12%) were not available.

The patients’ previous ocular surgeries and laser procedures are reported in Table 4.

The types of glaucoma of the patients enrolled in the study were primary open-angle glaucoma (87.2%, 238/273), pseudoexfoliative (6.6%, 18/273), primary angle closure glaucoma (2.6%, 7/273), uveitic (2.1%, 6/273), steroid-induced (1.1%, 3/273), and post-traumatic (0.4%, 1/273) glaucoma. 

The mean number of medications taken by the patients was 2.7 ± 0.9; however, 42.9% (117/273) of the patients used 3 active compounds, 2.5% (7/273) used 4 active compounds, and 16.1% (44/273) used 5 active compounds (including systemic carbonic anhydrase inhibitors). The patients’ drug regimens are represented in Table 5. Thirty-one percent of patients (85/273) were also using systemic carbonic anhydrase inhibitors.

**Figure 1:**
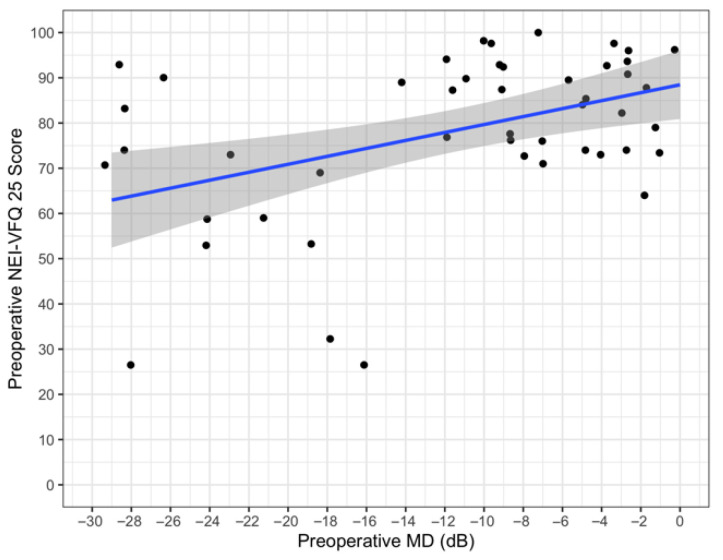
Preoperative NEI VFQ25 vs. preoperative MD.

The mean score of the NEI-VFQ 25 was 78 ± 18 (range 26.5–100), while the mean score of the GSS was 85 ± 14 (range 79–93), and only 60 patients competed the questionnaires. Linear regression analysis was performed at baseline between the baseline NEI-VFQ 25 score and the MD (Figure 1). The Spearman correlation coefficient was 0.42, with a 95% bootstrap CI: 0.07–0.60 (1000 bootstrap replications). The regression coefficient was 0.80 (*p* = 0.002). In our series, the patients with a worse MD value presented a lower NEI-VFQ score.

All the patients (273/273) underwent implantation preceded by a subconjunctival injection of 0.1 mL of mitomycin C (0.2 mg/mL), and 39.9% (109/273) of the implantations were performed in combination with cataract surgeries. The ophthalmic viscosurgical device used was cohesive in 90.11% of the cases (246/273), dispersive in 6.96% of the cases (19/273), and Duovisc^®^ (Alcon Laboratoires, Fort Worth, TX, USA) in 3.14% of patients (8/273). The surgeon’s position was mainly superior (178/273), but in 34.79% of the cases, it was temporal.

The implant was in a supero-nasal position in 91.58% of the cases (250/273), nasal in 4.39% of the cases (12/273), superior in 1.47% of the cases (4/273), supero-temporal in 1.47% of the cases (4/273), and temporal in 1.09% of the cases (3/273). The estimated depth was subconjunctival in 89.01% of the cases (243/273). Of the remaining cases, 7.3% (20/273) of the implants were judged as subtenonian and 3.66% (10/273) were judged as undeterminable. The XEN subconjunctival course immediately after implantation was linear in 86.45% (236/273) of the patients, curved in 1.47% (4/273), and undeterminable in 12.09% (33/273).

The implant was visible in the anterior chamber in 90.10% of the patients’ eyes (246/273). In 4.03% (11/273) of the patients, the XEN was visible in the anterior chamber only gonioscopically, and in 5.86% (16/273) of the patients, it was not visible at all.

Bleb formation was noted in 87.67% (239/273) of the patients; a flat bleb was observed in 12.45% (34/273) of the patients.

Intraoperative complications were seen in 39.93% of the cases (109/273), consisting of microscopic hyphema (73/273), mild hyphema (Grade I; 34/273), and conjunctival tears (2/273).

One week after surgery, 4/273 patients were treated with an active compound (3 patients with BB and 1 with PG) and 3/273 with two active compounds (BB and CAI). 

## 4. Discussion

When a new surgical intervention becomes available, questions regarding its effectiveness and safety in comparison with established techniques and questions regarding the ideal patient candidate are required to be answered to determine its best placement in the therapeutic algorithm of a specific disease. 

Disease progression, worsening QoL, and poor adherence should guide glaucoma specialists toward changing from a maximal tolerated medical treatment to selective laser trabeculoplasty, an MIGS, or a traditional surgery [10].

The XEN-GTR represents a national prospective glaucoma surgery registry in Italy, and it has been designed to prospectively collect multicentre, real-world data about the indications and use, alone or in combination with cataract surgery, of the XEN gel implant across a time-frame of 36 months. The XEN gel implant is a device designed for a minimally invasive surgical treatment of glaucoma and has been available in Italy since the year 2016. It represents the first ab-interno device designed for the subconjunctival filtration of the aqueous humour by acting as a shunt between the anterior chamber and the subconjunctival space, thus bypassing the outflow resistance of the trabecular meshwork and beyond [5,6,7,8,9]. 

From the data, is clear that the time of surgical indication may be adjusted based on the stage of the disease, the glaucoma therapy-related ocular surface disease, and the quality of life of the patient, and this has already been demonstrated in previous publications [14,15,16,17].

Among the potential advantages of the XEN gel implant over other established techniques such as trabeculectomy, there is a significantly reduced surgical time and a lack of conjunctival incisions, with potential advantages in terms of reduced inflammation and scarring [8,18,19,20,21,22,23].

The basal profile of the candidate patient for this kind of surgery within the Italian XEN-GTR is characterized by an average IOP of 24.0 mmHg with a wide variation spanning from 12 to 60 mmHg and by an average visual field defect, as expressed by the MD of −12 dB, and again with a wide variation spanning from −4 dB to −33 dB. Interestingly, the XEN gel implant in Italy has been proposed not only in patients with early to moderate glaucoma, but also in patients with severe glaucoma who are traditionally candidates for trabeculectomy.

Most of the enrolled population was represented by POAG (90%), and a smaller proportion of patients with secondary glaucoma was also enrolled and included pseudoexfoliative, uveitic, steroid-induced, and post-traumatic glaucoma. Considering that those categories of patients do not represent the primary indications of the XEN gel implant, it will be of great interest to explore the effectiveness and safety results in such challenging cases over the 36 months of planned follow-up for the study.

The preservation of QoL at a sustainable cost is the ultimate goal of any glaucoma treatment [10], and within the XEN-GTR, QoL measures such as the NEI VFQ-25 and GSS were recorded at baseline and will be monitored over time. It is noteworthy that collecting QoL data in our clinical practice represents an important limit because it takes time and our patients did not fully comprehend the significance of that information. Our preliminary data showed how patients with worse MD values presented lower NEI-VFQ scores. Therefore, it is fundamental to raise awareness about this topic and to stress the importance of collecting such data using questionnaires. 

It is interesting to highlight that the XEN was implanted as a unique intervention in 164 (60.1%) patients, of which 10 were phakic, and in which 109 (39.9%) received the implant in combination with cataract surgery, thus indicating either that the XEN as a solo procedure in phakic eyes is not a procedure of choice for most surgeons in Italy or that the coexistence of glaucoma and cataracts is highly prevalent. 

Despite the surgical technique for the XEN gel implant being standardized, some variations were undertaken within the XEN-GTR cohort, mainly regarding the implant location, in which a minority of cases (8.42%, 23/273) was different from the recommended supero-nasal quadrant and the type of ophthalmic viscosurgical device used, which was, in a majority of the cases, cohesive (90.11%).

When the XEN device is implanted, even by an expert surgeon, its depth with respect to the conjunctival and tenonian planes is known to be hardly predictable, even though a consistent surgical technique is demanded. Within the XEN-GTR cohort, in 89.01% of the cases, the depth of the implant was judged as subconjunctival, while in the remaining cases, it was judged as subtenonian. In 10 cases, it was judged as not determinable.

When the implant is released, its course may be found to be linear or curved under the tissues again, even if a consistent surgical technique is used. The linearity of the implant is likely to be related to the presence of subconjunctival tissue at the tip of the implant, which prevents its distension over the underlying tissue planes. Within the XEN-GTR, while immediately after implantation, the device was found to be clearly curved under the tissues in only 1.47% of the cases, in 12.09%, the course was undeterminable, most likely because of the deep subtenonian position of the implant itself. 

Again, despite a consistent implanting technique of a given surgeon, some implants may result in being clearly and directly visible in the anterior chamber, while other implants will be visible only gonioscopically or not visible at all. 

It is not known thus far whether the observed variations in the surgical technique or in the device placement within the eye tissues are able to influence the efficacy and safety outcomes over time, and this will be part of the associative analysis that will be performed after medium and long-term follow-up data become available

Maximum medical therapy is considered by most authors as the combination of three hypotensive agents or three drops per day, usually a prostaglandin analogue in association with a non-prostaglandin fixed combination [10]. In addition, an increased number of hypotensive topical medications with their preservatives has been shown to be associated with a reduced success rate of conventional filtering surgery [24].

The baseline results of the XEN-GTR indicate that, in Italy, 62% of the glaucoma surgical population is treated with three or more hypotensive agents, and thirty-one percent of patients (85/273) were also using systemic carbonic anhydrase inhibitors, suggesting a tendency to overtreat before undergoing surgery, with potential consequences on the outcome, which will be the objective of future investigations.

Indeed, compared to previous prospective studies, our study’s patients were enrolled without restrictions in terms of the type of glaucoma, baseline IOP value, visual field defect, or central corneal thickness [20,25,26,27,28,29,30], reflecting a real-world clinical scenario. This will allow for the evaluation of the XEN in a broader spectrum of patients with glaucoma, such as that seen in daily practice. 

Moreover, the design of the XEN-GTR as a surgical registry is aimed at making all data available for the scientific community with an interest in performing post-hoc analysis for clinical, economic, or health purposes.

## 5. Conclusions

In conclusion, based on the preliminary registered evidence, the typical Italian candidate for the XEN gel implant is a patient with POAG, on maximum medical therapy, and with moderate to severe visual field damage with no difference regarding the lens status. Comparing the Italian candidate with the recommended patients for the XEN gel implant, we can observe some relevant differences: most of the Italian patients scheduled for the implant were on a maximum medical therapy, which is different from a maximum tolerated medical therapy, and, ultimately, only 5% of the patients had a previous glaucoma surgery but not a MIGS. 

One year follow-up results regarding the efficacy and the safety of the implant will be published shortly, and they will integrate this preliminary evidence.

## Figures and Tables

**Table 1:** Reporting form recording the study visits and variables registered at baseline.

Study Visit	Time	Variables Registered	
		Demographic	Ophthalmological
Visit 0	1 to 4 weeks before surgery	Age Sex Ethnicity Systemic condition	Type of Glaucoma Lens state Previous surgery BCVA IOP Gonioscopy Medications VF RNFL OCT Indirect ophthalmoscopy ECC NEI-VFQ 25 GSS
Visit 1	Day of surgery	Type of surgery Surgeon position XEN position OVD XEN features Bleb formation Intra-operative complication Post-operative treatment	

OP, intraocular pressure; BCVA, best-corrected visual acuity; VF, visual field; RNFL OCT, retinal nerve fibre layer thickness optical coherence tomography; ECC, endothelial cell count; NEI-VFQ 25, National Eye Institute Visual Function Questionnaire-25; GSS, Glaucoma Symptoms Scale; OVD, ophthalmic viscosurgical device.

**Table 2:** Baseline ophthalmological data in the XEN-GTR.

Parameters	Mean Value ± SD	Range
BCVA (decimal)	0.53 ± 0.3	0.1–1
Spherical equivalent (dioptres)	−0.7 ± 2.5	−25.5–4.5
Anterior chamber depth (mm)	3.4 ± 0.8	2–6
Axial length (mm)	24.3 ± 2.4	20–35
Endothelial cell count (cell/mm^2^)	2108 ± 461	600–3076
Lens status, no. (%)	127 (46.5%) pseudophakic 145 (53.1%) phakic 1 (0.3%) aphakic	

EN-GTR, XEN Glaucoma Treatment Registry; BCVA, best-corrected visual acuity; SD, standard deviation.

**Table 3:** Baseline ophthalmological parameters describing the glaucoma condition of the enrolled patients in the XEN-GTR.

Parameters	Mean Value ± SD	Range
IOP (mmHg)	24 ± 6	12–60
Ophthalmoscopic cup/disc ratio	0.7 ± 0.14	0.3–1
CCT (µm)	523 ± 38	424–650
MD (dB)	−12.3 ± 8.6	−33–4
RNFL thickness (µm)	68 ± 19	0.63–120

OP, intraocular pressure; CCT, central corneal thickness; MD, mean deviation; RNFL, retinal nerve fibre layer; SD, standard deviation; XEN-GTR, XEN Glaucoma Treatment Registry.

**Table 4:** Previous ocular surgeries and laser procedures of the patients enrolled in the XEN-GTR (no. (%)).

Previous Ocular Surgeries and Laser Procedures	
None	141 (51.6%)
Cataract surgery	116 (42.5%)
Trabeculectomy	1 (0.4%)
Combined surgery (phacotrabeculectomy)	2 (0.7%)
Selective laser trabeculoplasty/argon laser trabeculoplasty	6 (2.2%)
Retinal detachment	1 (0.4%)
Other	5 (1.8%)
Not answered	1 (0.4%)

EN-GTR, XEN Glaucoma Treatment Registry.

**Table 5:** Treatment regimens of the patients enrolled in the XEN-GTR (no. (%)).

Compound	N (%)
BBA2	1 (0.3)
CAI	2 (0.7)
BBCAI preserved	3 (1.1)
PGBB preserved	4 (1.4)
A2CAI	4 (1.4)
PG preserved	5 (1.8)
BB	6 (2.2)
A2CAI + BBPG	7 (2.5)
A2CAI + PG	7 (2.5)
PG	15 (5.5)
BBCAI	19 (6.9)
PG + BBA2	23 (8.4)
PGBB	40 (14.6)
PG + BBCAI + A2	44 (16.1)
PG + BBCAI	87 (31.8)
NA	1 (0.3)
No medications	5 (1.8)

B, beta-blocker; PG, prostaglandin analogues; CAI, carbonic anhydrase inhibitors; A2, alfa2 adrenergic agonists; XEN-GTR, XEN Glaucoma Treatment Registry.

## Data Availability

The data presented in this study are available on request from the corresponding author after the authorization of the scientific committee of the Italian XEN Glaucoma Treatment Registry. The data are not publicly available because they are property of members of the Italian XEN Glaucoma Treatment Registry (XEN-GTR) and the study is still ongoing.
